# Determination of the vertical dimension occlusion – case report

**DOI:** 10.1080/07853890.2021.1896930

**Published:** 2021-09-28

**Authors:** S. Félix, M. João Barreto, A. Mariz de Almeida, A. Alves, P. Cebola, P. Maurício

**Affiliations:** a Instituto Universitário Egas Moniz (IUEM), Caparica, Portugal; b Centro de Investigação Interdisciplinar Egas Moniz (CiiEM), Egas Moniz Cooperativa de Ensino Superior, Caparica, Portugal

## Abstract

**Introduction:**

Despite the improvement in oral health care, tooth loss, for reasons associated with caries or periodontal disease, continues to be a reality in our population [1]. The planning of a total or a partial dental prosthesis is the key for the creation of a harmonious and natural relationship between the prosthetic structures, the tissues and the face, combining functional rehabilitation with aesthetics. The correct Vertical Dimension of Occlusion (VDO) is a key aspect in oral Rehabilitation [2].

**Materials and methods:**

A 49-year-old female patient attended the Oral Rehabilitation consultation with complaints about the aesthetic viability of her partial upper prosthesis with decreased VDO, which she had been using for about 20 years. The patient was advised to make a new prosthesis, not only to improve the aesthetics but also to increase the VDO to restore harmony at the soft tissue level. Clinically we started the usual protocoled oral rehabilitation procedure: 1. Clinical History, Preliminary Impressions, Profile teleradiography with the old prosthesis; 2. Definitive Impressions; 3. Intermaxillary recording, VDO recording using the Willis method; 4. Wax try in with phonetic and aesthetic test; cephalometric analysis; 5. Prosthesis delivery;

**Results:**

Our main objective was to measure and to establish a functional and ideal VDO. Considering the Wills Method, the 2/3 anterior facial height (AFH) was 67 mm, the patient prosthesis was 60 mm and the wax try in was 67 mm. In terms of cephalometry the angles related to VDO were lower compared the wax in and with the mean values in the prosthesis (i.e. Jaraback Analysis – Anterior Facial Height − 102° prosthesis 117°, wax in and mean value of 113°). We were able to safely assure the VDO not only from a soft tissue point of view, as well as phonetically, aesthetically and with cephalometry.

**Discussion and conclusions:**

The establishment of a correct VDO using different methods is crucial in the construction of the dental prosthesis, allowing a stable, comfortable and functional rehabilitation for the patient [1]. The Willis method, soft tissue and phonetics analysis, as well as cephalometric analysis, obtained good clinical results, similar to those achieved by other authors [1–3]. Orthlieb et al. [3] also confirmed that cephalometric measurements are important references in the measurement of VDO in partial and total edentulous patients. Total / Partial prosthesis continue to be a valid solution for the rehabilitation of partial edentulous patient both functionally as aesthetically. It is possible to determine the VDO, in a simple and scientifically correct manner, using if possible, a combination of the available techniques, in a partially edentulous patient.
